# Computer Programming: Should Medical Students Be Learning It?

**DOI:** 10.2196/11940

**Published:** 2019-03-22

**Authors:** Caroline E Morton, Susan F Smith, Tommy Lwin, Michael George, Matt Williams

**Affiliations:** 1 Medical Education Research Unit School of Medicine Imperial College London London United Kingdom; 2 Faculty of Medicine Imperial College London London United Kingdom; 3 Computational Oncology Lab Institute of Global Health Innovation Imperial College Healthcare NHS Trust London United Kingdom

**Keywords:** coding, medical education, undergraduate curriculum

## Abstract

**Background:**

The ability to construct simple computer programs (coding) is being progressively recognized as a life skill. Coding is now being taught to primary-school children worldwide, but current medical students usually lack coding skills, and current measures of computer literacy for medical students focus on the use of software and internet safety. There is a need to train a cohort of doctors who can both practice medicine and engage in the development of useful, innovative technologies to increase efficiency and adapt to the modern medical world.

**Objective:**

The aim of the study was to address the following questions: (1) is it possible to teach undergraduate medical students the basics of computer coding in a 2-day course? (2) how do students perceive the value of learning computer coding at medical school? and (3) do students see computer coding as an important skill for future doctors?

**Methods:**

We developed a short coding course to teach self-selected cohorts of medical students basic coding. The course included a 2-day introduction on writing software, discussion of computational thinking, and how to discuss projects with mainstream computer scientists, and it was followed on by a 3-week period of self-study during which students completed a project. We explored in focus groups (FGs) whether students thought that coding has a place in the undergraduate medical curriculum.

**Results:**

Our results demonstrate that medical students who were complete novices at coding could be taught enough to be able to create simple, usable clinical programs with 2 days of intensive teaching. In addition, 6 major themes emerged from the FGs: (1) making sense of coding, (2) developing the students’ skill set, (3) the value of coding in medicine, research, and business, (4) role of teaching coding in medical schools, (5) the concept of an enjoyable challenge, and (6) comments on the course design.

**Conclusions:**

Medical students can acquire usable coding skills in a weekend course. They valued the teaching and identified that, as well as gaining coding skills, they had acquired an understanding of its potential both for their own projects and in health care delivery and research. They considered that coding skills teaching should be offered as an optional part of the medical curriculum.

## Introduction

In recent years, there has been an increasing recognition that the ability to understand and construct simple computer programs (coding) is now a core skill and likely to be even more so in the future. This reflects the increasingly digital world in which we live and work and the growing need for more individuals to be able to engage with this type of technology. For example, the National Health Service (NHS) has recognized that when faced with an increasingly tight budget and time constraints, technology has the potential to help improve efficiency. Doctors and other health care professionals are in a unique position to identify problems that could be resolved or helped, in part, by technology. There have been numerous examples in the past few years of technology such as computer programs or specialist websites being used in clinical care [1-3] or research [4], and this is likely to continue to be the trend going forward. However, doctors and other health care professionals usually lack the understanding of which real-life problems could be resolved using computational approaches and very few have the ability to code the necessary software. Therefore, we need to increase the number of health care professionals who have some relevant expertise. At present, primary school children are now being taught how to code, but there will be a gap of at least ten years in the United Kingdom before a large number of students arrive at medical school already possessing these skills.

With this in mind, we developed an introductory *Coding for Medics* course to teach medical students basic programming and introduce them to the concepts of computational thinking. This is the starting point to bridge the gap between these future doctors and the technology industry and potentially facilitate greater collaboration between them.

In general, there are 2 main approaches to teaching computer coding [5-7]. The *coding* approach focuses on teaching practical programming skills to solve usual real-life problems. Examples include Software Carpentry [8], which teaches coding to researchers, New York University’s Code Academy [9] and Duke University’s Web-based courses on coding [10]. In contrast, the *theoretical* approach concentrates on computational thinking to teach the ability to break a complex problem into small, solvable components and automate parts or perhaps all of this using an algorithm. Examples include Massachusetts Institute of Technology's recent introduction of a minor course in Computer Sciences for noncomputer science majors [11] and Carnegie Mellon University’s Center for Computational Thinking [12]. All the courses mentioned here have significant overlap with each other in that they use relatively modern computer languages and teach a combination of theory and practice. However, they differ in whether they concentrate on teaching practical coding to solve problems or develop computational thinking. The *Coding for Medics* course described here was designed to primarily focus on practical coding skills with the minimal amount of theory needed to underpin these skills. This approach was chosen in the hope that students would develop practical skills to take away and that they could then apply the skills independently rather than learn from a purely theoretical approach.

There are only a couple of examples of doctors or medical students being taught programming, both in the last 2 years, which we believe indicate a growing interest in this area. A recent paper from the University of Toronto teaches Python in a weekly session that runs over 1 year [13]. A course in the Netherlands ran over 2 days and aimed at teaching app building to doctors with good success [14]. However, outside medicine, there have been a number of courses aimed at teaching coding and computational thinking to nonspecialists. For example, within our own institution of Imperial College London, the undergraduate biology students are taught bioinformatics and some of their biology and biochemistry courses involve using the computer language, Python, to manipulate protein structure files.

A precourse questionnaire to find out precourse coding experience was administered, and postcourse written anonymous feedback was collected. In addition, a qualitative study was designed to evaluate the program by documenting students’ achievements and exploring their perceptions of the value of coding in medical school. The aim of this study was to address the following questions:

1. Is it possible to teach undergraduate medical students the basics of computer coding in a 2-day course?
2. How do students perceive the value of learning computer coding at medical school?
3. Do students see computer coding as an important skill for future doctors?

## Methods

### Design and Delivery of The Coding for Medics Course

The *Coding for Medics* course was developed as a 2-day course, which aimed to take complete coding novices and develop them to a point at which they could design and write simple computer programs. The course was taught in the Python (Version 3) computer language as it is used for teaching introductory courses in other departments at Imperial College London because of its capacity to teach the underlying concepts and as it is freely available.

The learning objectives were structured around the simple elements of programming (see Textbox 1). There was also a small component on developing computational thinking. The course was structured with an initial 2 days of intensive teaching with 1 lead academic supported by teaching assistants (TAs) with a ratio of 1 TA to 6 students. The TAs were mostly other students from the Computer Science department of the same university, adopting the example of Stanford University, which drew TAs from its student population to teach introductory classes [15].

**The intended learning objectives of the *Coding for Medics* course.**

By the end of this short course students will be able to:

1. Install different programming languages
2. Describe and discuss the difference between major families of computer programming languages
3. Install and use an Integrated Development Environment (IDE)
4. Describe how to structure a problem to allow the application of computational techniques
5. Find and use online resources on programming
6. Choose appropriate data types for simple computations
7. Describe and discuss difference in different control structures
8. Implement and test different control structures
9. Control simple input and output from a program
10. Read data in from an external file
11. Simple error handling and debugging

Following the intensive 2-day introduction, participants were given 2 to 3 weeks during which they designed, wrote, and submitted a simple project in their private study time. They were supported by a private online forum and email during this period. Students wrote a half-page summary of their project, which included the background, the problem they wanted to tackle, and how they mapped this computationally. These were reviewed, and students attended a short, formative feedback session to review common theme and problems emerging from their projects. Students received their feedback and were able to revise certain concepts (such as functions) with the teaching staff. This session concluded with a talk signposting the next steps available to students. The course was deliberately kept short as we were aware that medical students particularly suffer from curriculum overload, and we wanted to identify the minimum amount of time that would provide the students with usable skills.

#### Course Participants

The course was free and open to medical students in any year of the undergraduate medicine program at Imperial College London. It was scheduled during holidays or weekends to avoid timetable clashes with compulsory medical school teaching.

### Study Design

A precourse questionnaire was distributed to students to gather basic demographics and to assess their previous experience of coding. At the end of the course, all participants were invited to give written feedback. This comprised open-ended, white-space questions exploring their views on the positive features of the course and what changes would improve future iterations. A written feedback on the course specifics was collected from students at the end of each iteration, and this was analyzed thematically. A voluntary FG took place after each iteration, the purpose of which was to determine students’ views on the potential value of coding in the undergraduate curriculum. FGs took place in Imperial College London Medical School in July 2015 and February 2016. FG participants were purposively sampled from the complete cohort of 33 course attendees from 2 courses. All course attendees were invited to participate, and 13 students took part (n=9, n=4). The FGs were conducted by a person who had not been involved in the development or teaching of the course or the original design of the research study. Qualitative data were analyzed thematically [16].

#### Ethics

The study received ethical approval from the local Medical Education Ethics Committee (MEEC1415-28).

## Results

### Student Demographics and Previous Experience

The precourse questionnaire was completed by 94% (31/33) of the participants. Their mean age was 22 years (SD 3.3), and there were slightly more males (58%, 19/33) than females. The students came from all years of the 6-year undergraduate medical program with years 1, 3, and 5 contributing the highest number of participants (8, 7, and 7, respectively). Most students reported having no programming experience before joining the course (87%, 27/31). In all, 85% of the students gave written feedback at the end of the course.

### Student Outcomes

A total of 25 participants (75%, 25/33) submitted projects that ranged in complexity from simple symptoms sorters to more complicated data analysis of a Web-based dataset. Students used the main components they had learned about in the course and applied it to a practical small project. For example, 1 student developed an analysis tool of a Web-based dataset on breast cancer. Although this was not formally assessed, the project summaries showed depth in understanding and application of computational thinking (eg, demonstrated by sensible mapping from the problem to the code), in addition to practical coding skills.

### Qualitative Data Analysis

A total of 6 major themes emerged from the qualitative data analysis of both FGs and written feedback:

1. Making sense of coding
2. Developing the students’ skill set
3. The value of coding in medicine, research, and business
4. Role of teaching coding in medical school
5. The concept of an enjoyable challenge
6. Comments on the course design

#### Making Sense of Coding

Students reported that a key part of the course was to develop an understanding of what coding is; they also reported that it broke their previously-held perceptions that coding is the preserve of the computer-obsessed or mathematically gifted. They reported surprise because of the fact that coding required only a computer and a free downloadable program to get started and that it could be done by any medical student, making it much more accessible than they had previously thought:

Anyone can do it as long as you get taught or teach yourself.

Student, First focus group (FG)

Before…I didn’t know where to start. We know [now] loosely how it fits in the world and ways to follow it and make it relevant to our own lives.

Student A, First FG

#### Developing the Student’s Skill Set

In line with the objective measures of their ability to perform simple projects, students felt that they had established some basic skills in coding that would form a solid starting point for developing further research skills in the future or developing some kind of innovative project (such as a health app). They recognized that they were not expert coders themselves but valued the opportunity to learn the basics and develop the language to be able to talk to expert coders for future collaborations, having understood what is feasible with code. Students also felt that having acquired the basics, it was useful to have the next steps highlighted for them so that they could develop their own skill set specific to their interests.

Interestingly, students also felt that they had developed new ways of thinking and problem solving, breaking large problems into small solvable components. They recognized that although this is a key skill when coding, it is also applicable to other aspects of their medical studies and research. Students felt that as coding was an extension of their logic, it helped them think about things in a more logical way, and this could be applied in clinical contexts. They felt that as a large part of Medicine is problem solving, learning basic computational thinking was beneficial:

I found the course quite useful in just understanding how to break things down into simple concepts.

Student A, second FG

I think that was probably the best part of the project for me. Hear what problems people have tackled with their code in ways I never even thought of even with my own project. I was still thinking very linearly.

Student B, second FG

I think it would be incredibly useful for me to have this coding knowledge so I can use it in research…I think having this base knowledge means I can build on it and in future perhaps go down the career path the TAs or the course lead had.

Student A, second FG

In terms of trying to figure out which problems are computable and which ones aren’t and realising that actually what you thought isn’t computable might as well be.

Student C, second FG

#### The Value of Coding in Medicine, Research, and Business

Students were able to imagine many different uses for coding in developing innovation in Medicine, research, and business. Many described *limitless possibilities* for what you could do with code if you were competent enough. They could see coding as having many potential applications including developing business technologies in health care, data manipulation, and analysis in research and for making efficiency savings in the NHS:

The other thing is that the course gave us an appreciation of how coding can help clinical research or lab research.

Student B, first FG

The only way to make the NHS better without spending more money is to make it more efficient and I think using innovations such as those that can be instigated by computer programs, those would be very useful. They would enrich the NHS and probably you on a personal level as well.

Student C, second FG

I don’t think it’s only just within science because coding is used to make apps and create technology which will…solve other business problems within medicine and outside of medicine.

Student C, first FG

...if we think something is feasible [to do with coding], it probably is.

Student D, first FG

#### Role of Teaching Coding in Medical School

Students felt that learning to code has a place in medical school, particularly learning that is based within a university with a high number of technological courses such as Imperial College London. They expressed surprise at the fact that it was not already widely on offer. They were clear that this course should remain optional as they were doubtful that all medical students would be sufficiently interested and engaged to complete it. They were also clear that coding competency is different from digital literacy and although all doctors should be digitally literate, not all doctors will need to be competent coders:

I think it’s odd that…where every other degree course does have coding involved throughout… medicine has never had it involved at all.

Student A, second FG

I wouldn’t force it on everyone because not everyone is going to want to do it… I know that it’s a really digital world out there and you are going to come across code but you might never need to code.

Student D, first FG

I’d quite like it to be a BSc on Bioinformatics or Computer Science.

Student F, first FG

If [the medical school] wants to teach us research skills, I think then this kind of coding should very much be part of it.

Student C, second FG

#### The Concept of an Enjoyable Challenge

Students reported that although difficult at times, with a lot to learn in a short period of time, it was fun to learn coding. In general, they reported that they enjoyed the course and enjoyed learning a new skill. They felt that it challenged them in a different way when compared with how they normally learn in medical school. The written feedback was largely positive, with students feeling that they had achieved something by the end of the course and had enjoyed themselves. The key negatives were the pace of the course and a steep learning curve:

I think a lot of medics would enjoy this because it’s problem solving and that’s kind of what we do in medicine, problem solving.

Student D, second FG

#### Course Specifics

Much of the written feedback received was about specific parts of the course such as individual sessions or topics. In general, students liked the task-orientated focus of the course and appreciated that they were able to try to write code as they went along and not only at the end of the session. They also reported that it was better when these tasks were relevant to clinical topics. They liked the informal nature of the course and indicated an enthusiastic teacher and a high TA-to-student ratio (1:6) were the keys to success as they would have struggled with the steep learning curve without that level of support:

I liked the fact that it moved very quickly on to a sort of hands-on approach and the fact we covered a large amount.

Written anonymous feedback, first course

The TAs were extremely useful – I wouldn’t have been able to do it without them.

Written anonymous feedback, first course

## Discussion

### Principal Achievements

We have demonstrated that medical students who were complete novices at coding could be taught enough to be able to create simple usable clinical programs with 2 days of intensive teaching. Therefore, it would be feasible to introduce teaching of basic computing skills without overloading the medical curriculum, particularly if it were to be introduced as an option. This is one of the first courses specifically designed to teach undergraduate medical students to code and is in contrast to the other published example [14], teaching students over an intense period of time rather than over 1 year. This demonstrates that there can be a variety of ways to get content into the curriculum. Interestingly, the other published course also uses Python and covers similar material [14].

The students identified that the course needed to be practical, with low student-to-TA ratio, and made relevant to clinical problems. Of note, out of original 33 students who completed this course, 2 have now gone on to work with the course lead on extended projects of their own. Moreover, 1 student is writing a program to transform and analyze routine publishable data on cancer outcomes and the other is helping to develop a software for a prognostic tool for oncology patients.

A wider consideration about teaching coding to medical students is about its value to future doctors. Technology is increasingly being used in the day-to-day work of a doctor and/or clinical researcher, for example, electronic health records, electronic prescribing, wearable technologies (such as heart rate and exercise monitors), medical devices, and telemedicine as well as a plethora of interactive health apps and websites. By completing this course, students are acquiring skills that allow them to interact and manipulate the digital tools needed to develop their own apps, websites, research tools, and innovative technologies; this goes far beyond the requirements for digital literacy stipulated by the General Medical Council [16]. It may be that the definition of computer literacy will change as more pupils leave school with coding skills and thus the average person becomes more and more skilled in using computers. This course provided a simple starting point for students to start developing skills in this area and potentially go on to develop their own projects.

Interestingly, and unexpectedly, students recognized that their own skill set had increased after the course by developing not only practical coding skills but also the ability to think computationally. They not only identified that breaking a large problem down into smaller solvable steps was key for coding but also identified that it had uses in other parts of their lives. This was also seen in the paper detailing the University of Toronto course, which found a similarly in the students’ thinking about the course improving their approach to solving problems [14]. This has direct application in research where a big research question is often addressed by breaking it down into a series of smaller studies. The fact that students felt that this way of thinking might help them with solving clinical problems was an unexpected gain from attending this course. Although this is a limited observation, it supports the idea that computational thinking teaches key abstract, transferrable thinking patterns; it would be worth exploring this concept in further studies designed for this purpose.

### Limitations

A limitation of this study is that this project was relatively small and therefore provides no information about the scalability of teaching coding en masse to medical students. If medical schools do want to teach coding to their students on a large scale, further studies would be needed. In addition, the students were a self-selected group who we might expect to be more enthusiastic about learning about coding than the average student. Another limitation is that we also do not have information about how much time students spent on self-study between the main course and submitting a project for formative feedback.

### Future Development

The feedback we have had from those on the course has led to us continuing to offer it and to additionally extend it to a new 10-week module in Computational Medicine, which is now offered as an option to all students in their BSc year. This has allowed us to ease the pace of the course and add new coding work to include R and databases. Using R to develop statistical programming skills is a key part of the BSc option and it complements the core coding skills teaching. The extended option also increases the amount of computational thinking and theory and introduces a new element to encourage students to develop their experience in presenting and discussing technical topics to their peers. The BSc option culminates in an extended project. We have also continued to offer the 2-day Coding for Medics, and applications continue to grow. It is offered 2 to 3 times per year, and we now have at least eighty places per iteration. Despite this, it is oversubscribed, which we feel demonstrates a real appetite for this among students. Now, we also aim to include about 25% postgraduate health care professionals or researchers per iteration of the course. In addition, former students of the Coding for Medics course have gone on to found a medical student coding society, which currently has 200 members.

### Conclusions

It is possible to teach medical students the basics of computer coding in only 2 days. Students considered that coding skills teaching should be offered as an optional part of the medical curriculum. Students are open to and value coding opportunities in medical school. They reported that it teaches computational thinking, which is a transferable skill (ie, breaking a larger problem into small solvable components), in addition to the practical skill of coding. They consider it to be an important skill for the future.

## Abbreviations

FG: focus group
NHS: National Health Service
TA: teaching assistant
